# Dysregulated IL-7/IL-7R-CD132 Axis and Intestinal Microsporidiosis in Crohn’s Disease

**DOI:** 10.3390/pathogens15040429

**Published:** 2026-04-16

**Authors:** Carolina Hurtado-Marcos, Fernando Izquierdo, Soledad Fenoy, Carmen del Águila, Jaume Pérez-Griera, Salvador Benlloch, Cirilo Amorós, Carlos García Ballesteros, Francisca López Chuliá, Juan Carlos Andreu-Ballester, Carmen Cuéllar

**Affiliations:** 1 Universidad CEU San Pablo, CEU Universities, Urbanización Montepríncipe, 28660 Boadilla del Monte, Spain; ferizqui@ceu.es (F.I.); sfenrod@ceu.es (S.F.); cagupue@ceu.es (C.d.Á.); 2 Laboratory Department, University Clinical Hospital, 46010 Valencia, Spain; perez.griera@gmail.com; 3 Digestive Departament, Arnau de Vilanova Hospital, 46015 Valencia, Spain; salvador.benllochperez@uchceu.es (S.B.); camorosg@icloud.com (C.A.); 4 Hematology Departament, Arnau de Vilanova Hospital, 46015 Valencia, Spain; garcia_carbar@gva.es (C.G.B.); lopez_frachu@gva.es (F.L.C.); 5 Foundation for the Promotion of Health and Biomedical Research in the Valencian Region, 46020 Benimaclet, Spain; jcandreuballester@outlook.com; 6 Department of Microbiology and Parasitology, Complutense University of Madrid, 28040 Madrid, Spain

**Keywords:** Crohn’s disease, *Encephalitozoon cuniculi*, CD132, caspase-3, γδ T cells, CD56^+^ lymphocytes, apoptosis, mucosal immunity, biomarkers, surgical outcomes

## Abstract

Crohn’s disease (CD) is frequently accompanied by T-cell lymphopenia and impaired mucosal immunity, conditions that may predispose to intestinal microsporidiosis by *Encephalitozoon cuniculi*. This prospective case–control study examined the interplay between IL-7/IL-7 receptor (IL-7R) signaling and anti-*E. cuniculi* immune responses in 50 CD patients and 50 matched healthy controls. Serum IL-7 and anti-*E. cuniculi* IgG, IgM, IgA and IgE were quantified by ELISA, while intestinal expression of IL-7, CD127 (IL-7Rα) and CD132 (IL-7Rγ) was assessed by RT-PCR. Protein levels of IL-7 and caspase-3 were evaluated by Western blot, and lymphocyte subsets and apoptosis by flow cytometry. CD patients showed reduced anti-*E. cuniculi* IgG and IgM levels but increased seropositivity, indicating compromised humoral quality despite greater exposure. Compared with controls, CD was associated with decreased serum IL-7, increased mucosal IL-7, downregulated CD132, and diminished caspase-3, suggesting a disrupted IL-7/IL-7R-apoptosis pathway. In CD, IgA- and IgE-skewed responses correlated differentially with caspase-3 and CD56^+^ γδ T cells, while *E. cuniculi* seropositivity independently predicted a shorter surgery-free interval. These findings identify a profound dysregulation of the IL-7/IL-7R-CD132-caspase-3 axis in CD and implicate *E. cuniculi* exposure as a potential marker of impaired mucosal immunity and adverse outcomes.

## 1. Introduction

Crohn’s disease (CD) is associated with a deficiency of T lymphocytes, particularly γδ and CD8^+^ subsets, and reduced serum interleukin-7 (IL-7) levels, correlating with increased susceptibility to intestinal microsporidiosis and an abnormal humoral response against *Encephalitozoon cuniculi*, characterized by elevated specific IgE but impaired IgG production. This serological pattern suggests incomplete immunization and potential parasite persistence [1,2,3]. Detection of *Encephalitozoon* DNA in approximately 30% of CD intestinal biopsies, contrasting with its absence in healthy controls, further supports that CD patients constitute a high-risk group for intestinal microsporidiosis and that *Encephalitozoon* spp. may contribute to disease pathogenesis [3].

Previous studies have demonstrated increased mucosal IL-7 expression, reduced caspase-3 activity, and markedly decreased expression of the common γ-chain (CD132) of the IL-7 receptor in the intestinal mucosa of CD patients [4,5]. These findings are linked to T-cell depletion, impaired mucosal immunity, and enhanced seroreactivity to *Anisakis simplex* antigens (IgG, IgM, IgA), with intestinal IgA responses correlating with disease activity [6]. Collectively, these data support a unified pathophysiological model in which a primary or acquired defect of the T-cell compartment and of IL-7/IL-7R (CD132) signaling in CD predisposes to opportunistic intestinal infections (*Encephalitozoon*, *Anisakis*) and leads to aberrant humoral responses, characterized by elevated IgE and dysregulated IgG/IgA production, that are insufficient to clear the pathogens [4,5,6].

Resolution of *Encephalitozoon* infection critically depends on CD8^+^ and γδ T lymphocytes [7,8,9,10,11,12,13,14,15,16,17,18]. Given that *Encephalitozoon* spp. might exploit the IL-7 deficiency and T-cell dysfunction observed in CD to persist within the intestinal mucosa, it becomes essential to investigate whether the IL-7/IL-7R-CD132 signaling axis, previously implicated in *Anisakis*-associated immune dysregulation, is also altered in *Encephalitozoon* infection among CD patients [1,6,19].

The present study aims to determine whether the IL-7/IL-7R-CD132 axis represents a shared mechanism of susceptibility to intestinal pathogens in CD, specifically microsporidia and helminths. Additionally, we sought to evaluate whether specific IL-7/IL-7R expression patterns, caspase-3 activity, and anti-*E. cuniculi* seroreactivity may serve as biomarkers of increased risk for opportunistic infection or a more aggressive disease course. This approach may contribute to identifying therapeutic strategies aimed at restoring IL-7/IL-7R signaling, with potential benefits for both intestinal immune homeostasis and pathogen control in CD.

## 2. Materials and Methods

### 2.1. Study Population

A prospective case–control study was conducted including 100 subjects: 50 patients diagnosed with CD and 50 age- and sex-matched healthy controls. Additionally, intestinal tissue samples from 20 CD patients and 20 healthy individuals were analyzed. CD diagnosis and classification followed Lennard-Jones criteria, integrating clinical, endoscopic, radiological, and histopathological findings [20]. Disease activity was determined using the Crohn’s Disease Activity Index (CDAI) [21]. Patients were stratified as follows: (1) Newly diagnosed: Active CD at presentation without previous treatment (≤24 h after therapy initiation); (2) Remission: Sustained CDAI < 150 for ≥12 months; (3) Active disease: CDAI > 150.

Control tissue biopsies were obtained from individuals undergoing colonoscopy for routine colorectal cancer screening, and all had normal endoscopic findings. Healthy controls had no history of recent vaccination within the previous 3 months, immunosuppression, autoimmune disease, or inflammatory disorders. Participants were recruited at Arnau de Vilanova Hospital (Valencia, Spain). The study was approved by the Research Committee of the Health Department of the Arnau University Hospital of Vilanova-Lliria (Valencia, Spain) and adhered to national and European ethical and data protection regulations. Written informed consent was obtained from all participants [1,4,6].

### 2.2. Tissue Sampling and Processing

Ileal and colonic biopsies (3–5 samples per subject) were collected via endoscopy or surgical resection. Samples were fixed in 10% neutral-buffered formalin for histological analysis or snap-frozen in liquid nitrogen for molecular and immunological studies. Frozen tissues were mechanically and enzymatically dissociated for cell isolation, using established protocols previously described. Single-cell suspensions were stained with fluorochrome-conjugated monoclonal antibodies for flow cytometric analysis of lymphocyte subsets [1,6].

### 2.3. Encephalitozoon Cuniculi Antigen and Serology

Antigenic extracts of *E. cuniculi* (USP-A1 strain) spores were prepared according to Águila et al. [22]. Specific antibodies in human sera were quantified by ELISA as previously reported [3,6,23,24]. Antibody reactivity was determined using HRP-conjugated secondary antibodies (BioSource International, Camarillo, CA, USA; INGENASA, Madrid, Spain; CALTAG Laboratories, Burlingame, CA, USA) and optical density readings at 490 nm.

### 2.4. Serum IL-7 Quantification

Serum interleukin-7 (IL-7) concentrations were measured in duplicate using a high-sensitivity ELISA (Quantikine^®^ HS IL-7, R&D Systems, HS750, Minneapolis, MN, USA), following the manufacturer’s protocol.

### 2.5. Cell Isolation and Flow Cytometry

Peripheral blood mononuclear cells (PBMCs) were isolated by density-gradient centrifugation (Lymphoprep™, Palex Medical, Barcelona, Spain). After washing, cells were resuspended in binding buffer containing calcium and stained for surface markers using monoclonal antibodies specific for TCRαβ, TCRγδ, CD3, CD4, CD8, CD19, CD56, CD5, and CD45 (Beckman Coulter, Brea, CA, USA). Data were acquired on a Navios flow cytometer and analyzed with Kaluza software. Cell apoptosis was evaluated using the Annexin V-FITC/7-AAD kit (Beckman Coulter). Figure S1 shows the gating strategy for T-cell subsets.

### 2.6. Gene Expression Analysis (IL-7, CD127, CD132)

Gene expression was analyzed by RT-qPCR. Total RNA (1 µg) was isolated from intestinal tissues with TRIzol^®^ Reagent (Ambion, Life Technologies, Carlsbad, CA, USA) and reverse-transcribed using RevertAid H Minus First-Strand cDNA Synthesis Kit (Thermo Fisher, Waltham, MA, USA). Quantitative PCR was performed using SYBR Green chemistry in a GeneAmp 5700 system (Applied Biosystems, Foster City, CA, USA). GAPDH was used as the reference gene, and relative expression was calculated using the 2^−ΔΔCT^ method. Primer sequences are provided in our previous works [4,6].

### 2.7. Protein Expression of IL-7 and Caspase-3

Protein extracts were obtained using the Trizol/Guanidine method (Ambion, SIGMA, Austin, TX, USA). Equal amounts (20 µg) were electrophoresed on 12% SDS-PAGE gels and transferred to nitrocellulose membranes. Membranes were incubated with primary antibodies against IL-7 and caspase-3 [4,6], followed by HRP-conjugated secondary antibodies (Merck, Darmstadt, Germany). Immunoreactive bands were visualized using ECL reagents (Amersham, Little Chalfont, UK) and quantified by densitometry (ImageJ 1.51). Actin served as the loading control (Figure S2).

### 2.8. Statistical Analysis

Data analysis was performed using GraphPad Prism 8.0.0 (GraphPad Software, San Diego, CA, USA) and IBM SPSS Statistics 19.0 (SPSS Inc., Chicago, IL, USA). The Mann–Whitney U test was applied for non-parametric comparisons between groups. Chi-square or Fisher’s exact tests were used for categorical data. Correlations among IL-7 expression, caspase-3 activity, and antibody levels were assessed with Spearman’s rank test. Kaplan–Meier analysis was used to assess surgical relapse-free survival according to anti-*E. cuniculi* immunoglobulin serostatus in patients with CD. Cox proportional hazards regression was performed to report hazard ratios (HRs) and 95% confidence intervals (CIs) for both univariate and multivariate models. Surgical relapse was defined as intestinal surgery performed because of disease-related complications. Statistical significance for clinical scenarios was assessed using ANOVA with the Bonferroni post hoc test. A *p* value of <0.05 was considered statistically significant.

## 3. Results

Table 1 summarizes the demographic and clinical characteristics of the CD patients.

### 3.1. Anti-Encephalitozoon cuniculi Antibodies in Patients with Crohn Disease and Healthy Subjects

**Figure 1:**
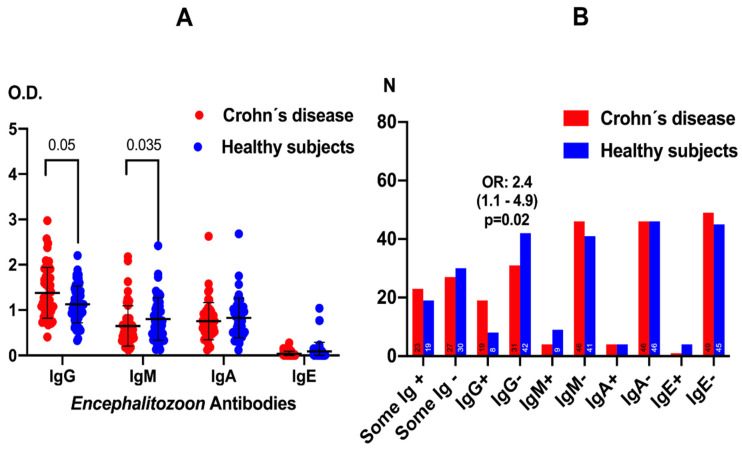
Levels of anti-*Encephalitozoon cuniculi* antibodies in patients with Crohn’s disease and healthy controls. Data are presented as means ± standard deviation to illustrate group variability. Statistical comparisons were performed using the Mann–Whitney U test for continuous variables (Panel (**A**)), and the Chi-square or Fisher’s exact tests for categorical data (Panel (**B**)).

Patients with CD displayed a differential antibody pattern against *E. cuniculi* compared with healthy subjects, both in antibody titers and in the frequency of seroreactivity (Figure 1). In terms of antibody levels (Figure 1A), mean IgG and IgM anti-*E. cuniculi* optical densities were significantly lower in CD patients than in controls (*p* = 0.05 and *p* = 0.035, respectively, Mann–Whitney U test), whereas IgA and IgE levels did not differ significantly between groups. Although a wide dispersion was observed in both cohorts, healthy subjects tended to cluster at higher IgG and IgM values, suggesting a more robust humoral response to the microsporidium in the control population than in CD patients.

When the frequency of seroreactivity was analyzed (Figure 1B), the number of individuals with at least one positive antibody isotype against *E. cuniculi* (“Some Ig+”) was higher in the CD group than among healthy subjects, with an odds ratio of 2.4 (95% CI 1.1–4.9, *p* = 0.02; chi-square/Fisher’s exact test). This higher proportion of seropositive individuals in CD, despite their lower IgG and IgM levels, indicates more frequent exposure and seroreactivity to *E. cuniculi* in these patients, consistent with an increased risk of intestinal microsporidiosis and a qualitatively altered adaptive immune response.

### 3.2. Assessment of IL-7, Its Receptor and Caspase-3 According to Positivity of Anti-Encephalitozoon cuniculi Antibodies

In patients with CD, the presence of anti-*E. cuniculi* antibodies was globally associated with similar tissue levels of IL-7 and caspase-3, as well as comparable expression of CD132 and CD127 receptors, compared to seronegative patients, with no statistically significant differences between groups.

**Figure 2:**
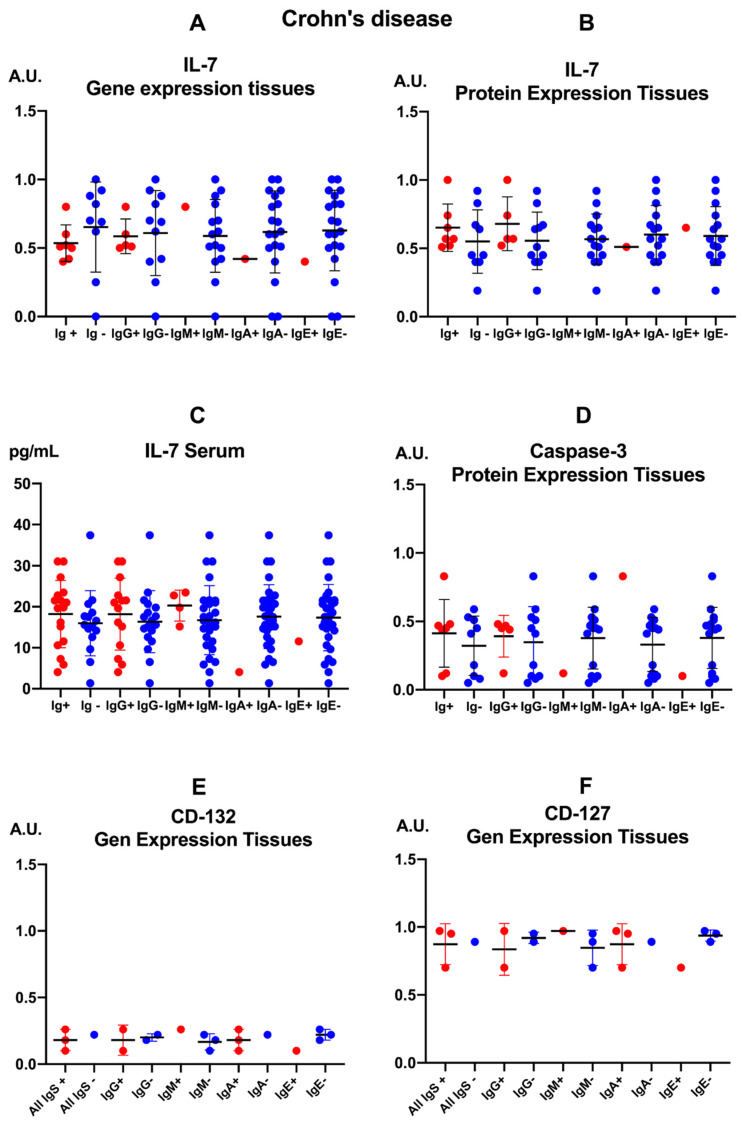
Immune parameters in patients with Crohn’s disease (CD) categorized according to the presence (+) or absence (-) of anti-*Encephalitozoon cuniculi* antibodies. The figure is organized into panels showing: interleukin-7 (IL-7) gene expression (**A**); IL-7 protein levels in intestinal tissue (**B**); serum IL-7 concentrations (**C**); caspase-3 protein levels in intestinal tissue (**D**); and CD132 and CD127 receptor subunit gene expression ((**E**) and (**F**), respectively). Data are shown as means with double T-bars representing standard deviations. Statistical significance was assessed using the Mann–Whitney U test.

In intestinal biopsies, both IL-7 gene expression (Figure 2A) and tissue protein concentrations (Figure 2B) showed closely overlapping mean values between Ig+ and Ig- groups across different anti-*E. cuniculi* isotypes, without clear shifts toward higher or lower levels in either subgroup. Serum IL-7 concentrations (Figure 2C) were similarly distributed between seropositive and seronegative patients, with comparable means across all isotype subgroups, indicating that seropositivity to the parasite does not coincide with appreciable systemic alterations in IL-7 in this cohort.

Tissue caspase-3 levels (Figure 2D), a marker of apoptosis, remained similarly distributed between patients with and without anti-*E. cuniculi* antibodies, showing no consistent trends toward increases or decreases associated with seroreactivity to the microsporidium. CD132 (γc subunit) gene expression in intestinal tissue (Figure 2E) was low in both groups, with means virtually overlapping across different seroreactivity patterns, suggesting that the CD132 deficiency described in CD is not modulated by anti-*E. cuniculi* antibodies. Likewise, tissue CD127 expression (Figure 2F) remained elevated and homogeneous across Ig+ and Ig- subgroups for each isotype, without relevant changes linked to the humoral response to the parasite, pointing to a dissociation between intestinal microsporidiosis seropositivity and dysregulation of the IL-7/IL-7R-CD132 axis in these patients.

**Figure 3:**
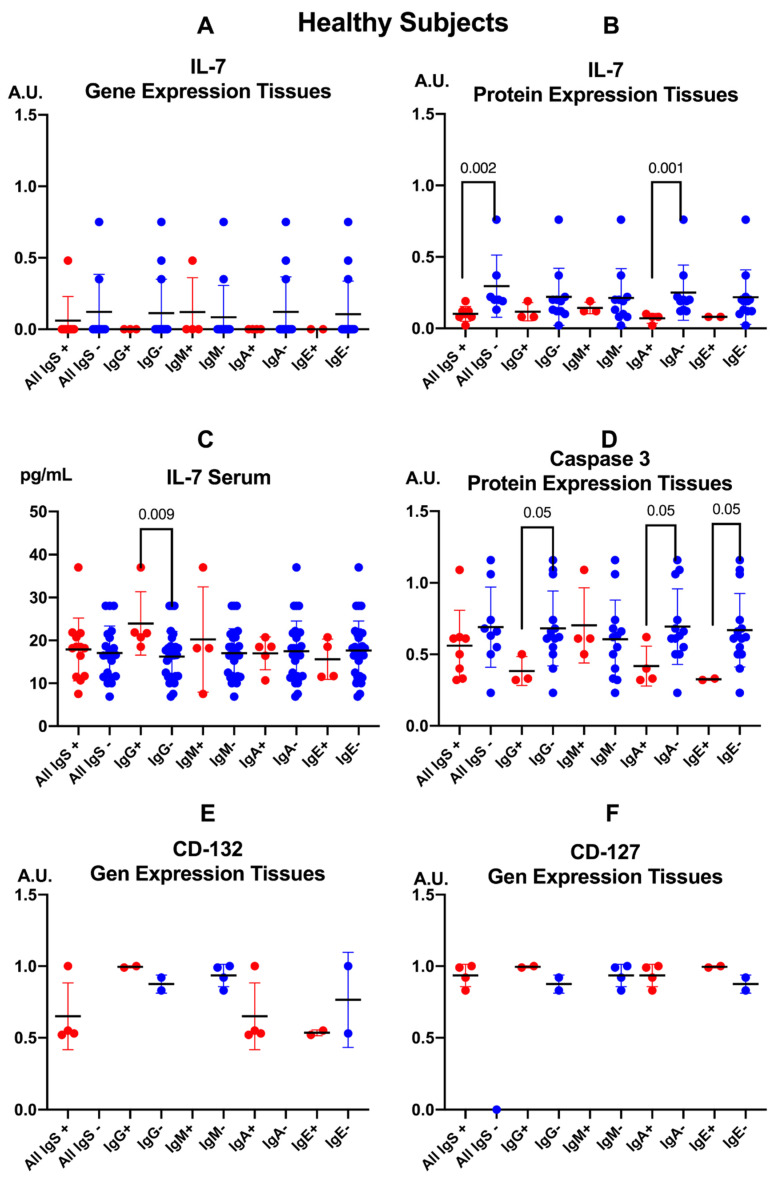
Immune parameters in healthy control individuals categorized according to the presence (+) or absence (-) of anti-*Encephalitozoon cuniculi* antibodies. The panels depict: (**A**) IL7 gene expression; (**B**) IL-7 protein levels in tissues; (**C**) serum IL-7 concentrations; (**D**) caspase-3 protein levels in tissues; and gene expression of IL-7 receptor subunits, (**E**) CD132 and (**F**) CD127. Data are presented as mean values with double T-bars indicating the standard deviation. Statistical significance was determined using the Mann–Whitney U test.

In healthy controls, the presence of anti-*E. cuniculi* antibodies is associated with selective activation of the IL-7/IL-7R-CD132 axis at the tissue level, without clear changes in IL-7 or its coreceptor gene expression (Figure 3).

The gene expression of IL-7 in intestinal tissue (Figure 3A) remained low, with no apparent differences between individuals positive or negative for anti-*E. cuniculi* immunoglobulins of any serum class (IgG, IgM, IgA, or IgE). In contrast, tissue IL-7 protein levels (Figure 3B) were significantly higher in subjects with detectable anti-*E. cuniculi* antibodies compared to seronegative individuals (All Igs+ vs. All Igs-, *p* = 0.002), and particularly in those positive for specific anti-*Encephalitozoon* IgA compared to IgA-negative counterparts (IgA+ vs. IgA-, *p* = 0.001). This finding suggests a local “overexpression” of IL-7 associated with sensitization to the parasite. In serum (Figure 3C), IL-7 concentrations were comparable across all subgroups, although individuals positive for anti-*E. cuniculi* IgG exhibited significantly higher levels than IgG-negative subjects (*p* = 0.009), indicating a systemic modulation of IL-7 linked to an IgG-mediated humoral response.

Intestinal caspase-3 protein expression (Figure 3D) tended to be higher in individuals with anti-*E. cuniculi* antibodies, reaching statistical significance in the “All Igs+” vs. “All Igs-“ comparison (*p* = 0.05) and in subjects positive for specific IgG, IgM, and IgE classes relative to their seronegative counterparts (*p* = 0.05 for each).

The gene expression of CD132 in intestinal tissue (Figure 3E) was relatively homogeneous, showing no marked differences between individuals with or without specific antibodies, whether analyzed collectively or by isotype. Likewise, CD127 expression (Figure 3F) remained essentially constant irrespective of *E. cuniculi* serostatus. These findings indicate that, in healthy controls, modulation of the IL-7 axis in response to parasite sensitization occurs primarily through altered production of IL-7 and caspase-3, rather than through changes in the expression of their coreceptors (Table A1, Table A2, Table A3 and Table A4).

### 3.3. Correlations Caspase-3, IL-7 and CD3^+^ CD56^+^ γδ T-Cell Subsets with Anti-Encephalitozoon cuniculi Antibodies

**Figure 4:**
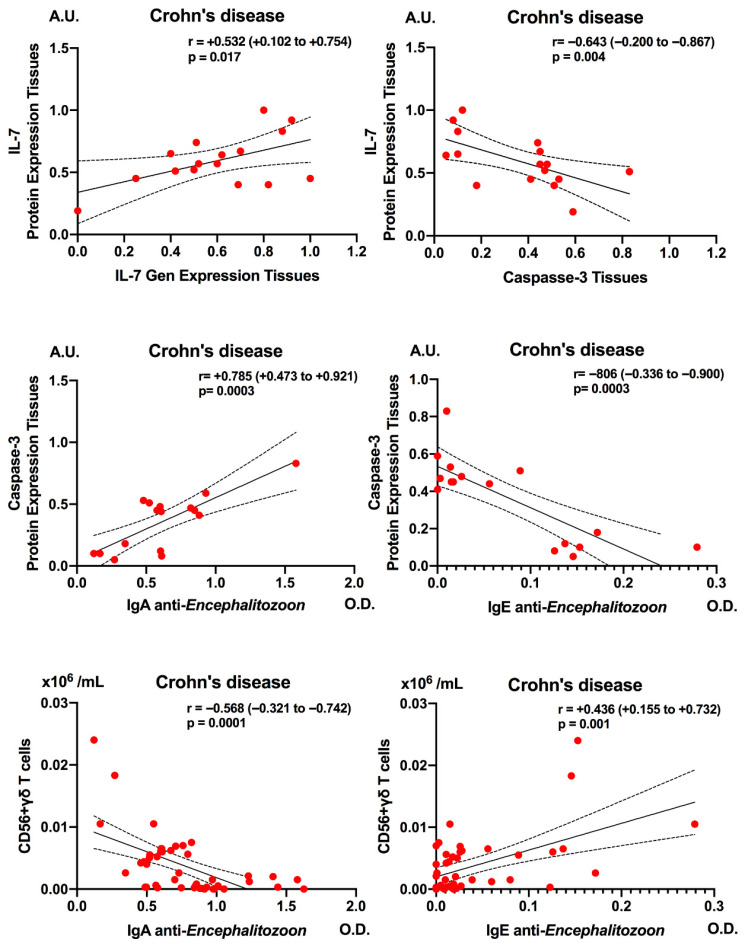
Correlation analysis between caspase-3 levels and CD56^+^ γδ T cells with IgA and IgE anti-*Encephalitozoon cuniculi* antibodies in patients with Crohn’s disease. Correlations were assessed using Spearman’s rank correlation test and are expressed as r values with 95% confidence intervals. The solid line indicates the linear regression line. Dashed lines represent the 95% confidence interval for the regression line.

In patients with CD, Figure 4 illustrates that dysregulation of the IL-7/IL-7R-CD132 axis and seroreactivity to *E. cuniculi* are associated with coordinated changes in tissue apoptosis (caspase-3) and circulating CD56^+^ γδ T lymphocytes. As demonstrated in our previous studies, a moderate positive correlation was observed between IL-7 gene expression in intestinal mucosa and tissue IL-7 protein levels (r = +0.532; *p* = 0.017), indicating that higher transcriptional activity translates into increased local IL-7 protein production. Moreover, tissue IL-7 levels of protein expression correlated inversely with caspase-3 protein expression (r = −0.643; *p* = 0.004), suggesting that local IL-7 upregulation is associated with reduced activation of the effector apoptotic pathway in the intestinal mucosa of these patients [4].

In the upper row of Figure 4 in the present study, anti-*E. cuniculi* IgA levels correlated positively with tissue caspase-3 concentrations (r = +0.785; *p* = 0.0003), whereas anti-*E. cuniculi* IgE values showed a strong negative correlation with caspase-3 (r = −0.806; *p* = 0.0003).

In the lower row of Figure 4, the panels illustrate that anti-*E. cuniculi* IgA levels correlated negatively with the frequency of circulating CD56^+^ γδ T lymphocytes (r = −0.568; *p* = 0.0001), while anti-*E. cuniculi* IgE values correlated positively with this subset (r = +0.436; *p* = 0.001).

Taken together, these correlations link dysregulation of the IL-7/IL-7R-CD132 axis and mucosal apoptosis with distinct patterns of humoral immune response and the relative depletion or expansion of CD56^+^ γδ T lymphocytes, which may contribute to increased susceptibility to intestinal microsporidiosis and to chronic inflammation in CD.

**Figure 5:**
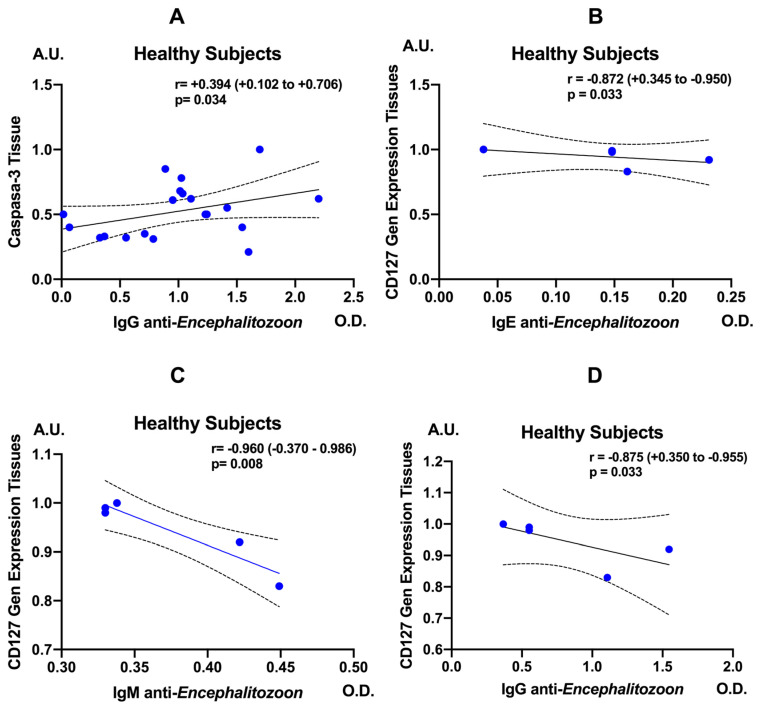
Correlation analysis between caspase-3 levels (**A**) and CD127 gene expression with IgE (**B**), IgM (**C**), and IgG (**D**) anti-*Encephalitozoon cuniculi* antibodies in healthy subjects. Correlations were evaluated using Spearman’s rank correlation test and are expressed as r values with 95% confidence intervals. The solid line indicates the linear regression line. Dashed lines represent the 95% confidence interval for the regression line.

In healthy controls, anti-*E. cuniculi* IgG levels showed a moderate positive correlation with tissue caspase-3 expression (r = 0.394; 95% CI, 0.102–0.706; *p* = 0.034), suggesting that a stronger IgG response to the parasite is accompanied by enhanced activation of apoptotic pathways (Figure 5A). Anti-*E. cuniculi* IgE levels correlated negatively and significantly with CD127 gene expression in intestinal tissue (r = −0.872; 95% CI, 0.345–−0.950; *p* = 0.033), indicating that elevated IgE responses are associated with reduced availability of the IL-7 receptor (Figure 5B–D). Consistently, a strong inverse correlation was also observed between anti-*E. cuniculi* IgM and CD127 (r = −0.960; 95% CI, −0.370–−0.986; *p* = 0.008), as well as between anti-*E. cuniculi* IgG and CD127 (r = −0.875; 95% CI, 0.350–−0.955; *p* = 0.033).

### 3.4. Positivity for Anti-Encephalitozoon cuniculi Immunoglobulins and Surgical Events in Patients with Crohn’s Disease

**Figure 6:**
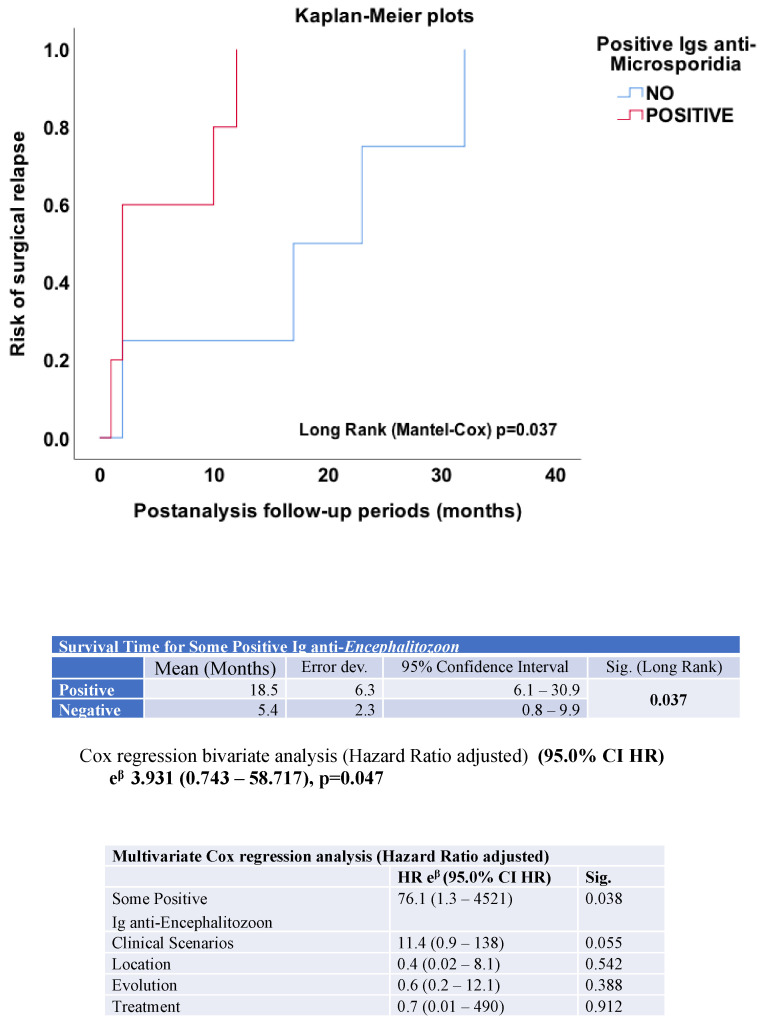
Kaplan–Meier analysis of surgical relapse-free survival according to anti-*Encephalitozoon cuniculi* immunoglobulin serostatus in patients with Crohn’s disease. Seropositive patients exhibited significantly shorter surgical relapse-free survival compared to seronegative patients (*p* < 0.05, log-rank test). Bivariate and multivariate Cox proportional hazards analyses were performed.

Positivity for anti-*E. cuniculi* immunoglobulins was associated with a significantly shorter time to surgical events in patients with CD, indicating an increased early hazard of surgical complications. Of the 50 patients with CD included in the study, 15 (30%) required surgical intervention during follow-up (five for intestinal stenosis and four for fistulizing disease). Anti-*E. cuniculi* immunoglobulins were analyzed to assess their association with the risk of surgery. The relationship between antibody positivity and the need for surgical intervention was evaluated using bivariate Cox regression analysis, and results were expressed as adjusted hazard ratios. The Kaplan–Meier curve illustrating the risk of surgical relapse in patients with positive anti-*E. cuniculi* antibodies is shown in Figure 6.

In the Kaplan–Meier analysis, patients seronegative for anti-*E. cuniculi* antibodies exhibited a longer surgery-free survival during post-analysis follow-up, whereas seropositive individuals experienced earlier surgical events, with a significant separation between the curves (log-rank test, *p* = 0.037). The estimated mean time to surgery indicated a markedly shorter surgery-free interval in seropositive patients (5.4 months; 95% CI: 0.8–9.9) compared with seronegative ones (18.5 months; 95% CI: 6.1–30.9), consistent with a less favorable clinical course in the group with intestinal microsporidiosis. The bivariate Cox regression analysis confirmed this association, showing an overall significant model (chi-square = 3.931, *p* = 0.047) and revealing that seropositivity to *Encephalitozoon* behaves as an independent risk factor for early surgical relapse in CD.

In the time-to-event analysis, CD patients seropositive for anti-*E. cuniculi* immunoglobulins demonstrated a significantly increased hazard and reduced time to surgical occurrence compared with seronegative patients.

### 3.5. Anti-Encephalitozoon cuniculi Immunoglobulins According to Clinical Scenarios

**Figure 7:**
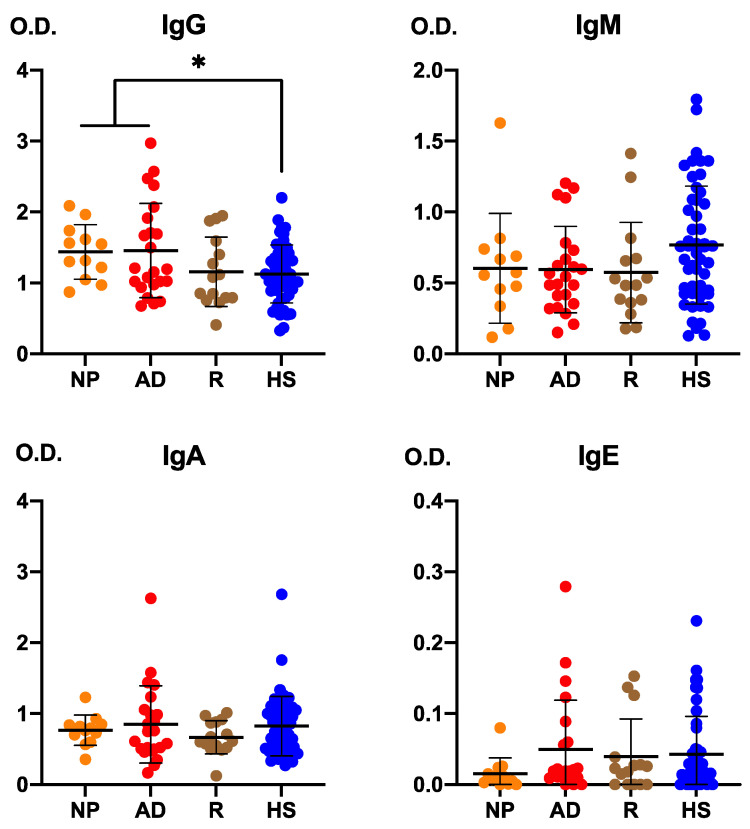
Anti-*Encephalitozoon cuniculi* immunoglobulin levels according to clinical scenarios (NP: newly diagnosed; AD: active disease; R: remission; HS: healthy subjects). Data are presented as mean values ± standard deviation (double T-bars). Statistical significance was determined using one-way ANOVA with Bonferroni post hoc test (*p* < 0.05). * *p* < 0.05.

Figure 7 shows anti-*E. cuniculi* IgG levels according to clinical scenarios. Anti-*E. cuniculi* IgG levels were significantly higher in patients with newly diagnosed and active disease compared to those in remission and healthy subjects (*p* < 0.05).

### 3.6. Anti-Encephalitozoon cuniculi Immunoglobulins According to Treatment Status

Analysis of anti-*E. cuniculi*-specific antibodies according to treatment status revealed no significant differences between treated and untreated patients (Table A5; multivariate Cox regression: HR = 0.7, 95% CI: 0.01–490, *p* = 0.912).

## 4. Discussion

Crohn’s disease is characterized by chronic intestinal inflammation and a profound disturbance of T-cell homeostasis in which IL-7/IL-7R signaling has emerged as a key regulator of lymphocyte survival, metabolism and mucosal immunity [25,26,27]. Genetic and functional alterations of the IL-7R pathway, including downregulation of the common γ-chain CD132, have been reported in CD and linked to γδ T-cell deficiency and impaired effector function. Microsporidia of the genus *Encephalitozoon* are obligate intracellular parasites whose control critically depends on robust CD8 and γδ T-cell responses, as shown in experimental *E. cuniculi* infection models [7,8,9,10,11,12,13,14,15,16,17,18]. Together, these observations provide a biologically plausible framework in which a dysfunctional IL-7/IL-7R-CD132 axis could contribute to chronic intestinal inflammation and may be associated with altered host responses to intestinal opportunists such as *Encephalitozoon* in CD [1,6,28,29].

Our current data extend previous observations in CD by demonstrating an altered humoral response to *Encephalitozoon* and a dysregulated IL-7/IL-7R-CD132-caspase-3 circuit both systemically and in the intestinal mucosa. In line with earlier work describing increased anti-*E. cuniculi* IgE but impaired IgG responses and frequent detection of *Encephalitozoon* in intestinal tissue from CD patients, we observed elevated anti-*Encephalitozoon* IgE and relatively reduced or inefficient IgG levels in CD compared with healthy controls, consistent with an incomplete or “primary-like” response that may favor intracellular persistence [3]. At the same time, CD patients showed decreased serum IL-7, but increased mucosal IL-7 expression accompanied by marked downregulation of IL-7R components (including CD132) and reduced caspase-3, indicating an uncoupling between IL-7 production and effective receptor-mediated signaling in the inflamed gut [4]. This pattern suggests a mucosal environment in which IL-7 is locally upregulated, yet fails to deliver appropriate survival and apoptotic cues to T cells because of receptor insufficiency, thereby promoting the accumulation of dysfunctional lymphocytes and the persistence of *Encephalitozoon* in the intestinal mucosa [30].

A key conceptual advance of this work is the proposal of *Encephalitozoon* as an “inverse model” to *A. simplex* within the same IL-7/IL-7R-CD132 axis. In CD, *A. simplex* infection is associated with enhanced seroreactivity, tissue overexpression of IL-7, reduced caspase-3 and downregulation of CD132, linking defective IL-7/IL-7R signaling to γδ T-cell depletion, altered apoptosis and granulomatous Th2-skewed inflammation [6]. In contrast, *Encephalitozoon* are intracellular pathogens that appear to exploit T-cell lymphopenia and IL-7/IL-7R defects to persist within macrophages and mucosal tissues, eliciting a skewed humoral response characterized by high IgE and low or functionally poor IgG. Studying *E. cuniculi* thus allows a direct comparison between an obligate intracellular parasite that capitalizes on T-cell depletion and impaired IL-7 signaling for persistence, and a tissue/extracellular helminth that induces granulomas and strong Th2 responses but converges on the same defective IL-7/IL-7R-CD132 pathway in CD. This convergence supports the notion that disruption of IL-7-dependent homeostatic circuits may represent a shared mechanistic basis for susceptibility to diverse intestinal opportunists in CD [9].

Within the CD cohort, seropositivity to *E. cuniculi* identified a subgroup of patients with a more pronounced immunological imbalance, characterized by higher anti-*Encephalitozoon* antibody levels, altered serum IL-7 concentrations and more marked changes in mucosal IL-7, IL-7R/CD132 and caspase-3 expression. These patients exhibited a deeper disruption of IL-7/IL-7R signaling and apoptosis pathways than seronegative CD patients, suggesting that mounting a humoral response to *Encephalitozoon* occurs in the context of a particularly dysfunctional IL-7-caspase-3 axis. This is consistent with experimental data showing that optimal immunity to *E. cuniculi* relies on early γδ T-cell responses that prime protective CD8 T-cell cytotoxicity, cell populations known to be critically dependent on intact IL-7R signaling and CD132 expression for survival and homeostatic proliferation. Our findings therefore support a model in which IL-7/IL-7R-CD132 dysregulation simultaneously constrains γδ and CD8 T cell-mediated clearance and skews humoral responses towards IgE-dominated or incomplete profiles, together favoring *Encephalitozoon* persistence and chronic mucosal inflammation in CD [10].

The observed relationships between anti-*E. cuniculi* antibodies, mucosal caspase-3 and γδ T-cell subsets further reinforce the functional coupling of IL-7 signaling, apoptosis and immune surveillance. In CD patients, IgA responses to *E. cuniculi* were associated with higher caspase-3 expression in intestinal tissue, whereas IgE-dominated responses correlated with reduced caspase-3, indicating divergent immune profiles against the same pathogen, one linked to increased apoptosis and potentially better control, the other to apoptosis impairment and persistence. These patterns align with the known role of IL-7 in tightly regulating T-cell apoptosis through modulation of pro- and anti-apoptotic molecules and maintenance of metabolic fitness. Moreover, the association between anti-*Encephalitozoon* antibodies and CD56^+^ γδ T-cell frequencies in CD indicates that both humoral responses and innate-like T-cell homeostasis are integrated within this IL-7-caspase-3-regulated network, in agreement with prior work linking CD132 deficiency to γδ T-cell depletion in CD tissues. In healthy subjects, seropositivity to E. cuniculi was accompanied by increased mucosal IL-7 and caspase-3 and reduced CD127 expression, suggesting a coordinated IL-7-caspase-3 response that enhances apoptotic clearance and immune surveillance without driving chronic inflammation. Together, these findings indicate that the IL-7/IL-7R-CD132 axis not only shapes T-cell survival, but also calibrates the balance between effective pathogen control and tissue damage via apoptosis in microsporidiosis [31].

Our results also have clinical implications, as seropositivity for anti-*Encephalitozoon* immunoglobulins was associated with a higher risk of surgical relapse and a shorter surgery-free interval in CD. This association suggests that anti-*E. cuniculi* antibodies may serve as a serological marker identifying CD patients with more severe mucosal immune dysfunction and a higher propensity for early surgical complications, complementing existing biomarkers used to stratify disease course and treatment response. In line with studies showing that heightened IL-7R pathway activity in intestinal tissue correlates with refractoriness to anti-TNF therapy and supports the persistence of pathogenic T cells in IBD, our findings point to IL-7/IL-7R-CD132 as a candidate biomarker of susceptibility to intestinal opportunists and of aggressive disease behavior in CD. Whether targeted modulation of IL-7R signaling, either to restore balanced T-cell homeostasis and apoptosis or to limit excessive IL-7R activity in specific disease stages, could improve control of opportunistic infections while attenuating chronic inflammation warrants further investigation in translational and interventional studies [32]. This study has several limitations that should be considered when interpreting the results. The moderate sample size from a single center may limit generalizability and preclude detailed subgroup analyses (e.g., by treatment class, disease location, or *Encephalitozoon* parasitological burden). The cross-sectional design precludes formal causal inference regarding the relationships between IL-7/IL-7R-CD132 alterations, *Encephalitozoon* serostatus, and disease progression. Moreover, anti-*Encephalitozoon* antibody detection indicates prior exposure rather than active infection or tissue parasite burden.

We also did not functionally dissect IL-7R signaling in specific T-cell subsets or assess *Encephalitozoon* effects on epithelial or myeloid cells, which represent additional potential targets of IL-7 in the intestinal microenvironment. Future longitudinal studies should integrate serial serology, tissue PCR detection of microsporidia, detailed phenotyping of γδ and CD8 T cells, and functional assays of IL-7R signaling and apoptosis to establish temporal relationships and mechanistic causality [30].

## 5. Conclusions

Our findings demonstrate that CD patients exhibit an altered humoral response to *E. cuniculi* and a profoundly dysregulated IL-7/IL-7R-CD132-caspase-3 axis, especially in those seropositive for *Encephalitozoon*, who show deeper defects in mucosal signaling and an increased risk of early surgical complications. These data support a model in which disruption of IL-7-dependent pathways creates a permissive environment for intestinal opportunists by impairing γδ and CD8 T cell-mediated surveillance, skewing antibody responses and altering apoptosis, thereby reinforcing chronic inflammation. By integrating microsporidiosis into the same IL-7/IL-7R-CD132 circuit previously implicated in CD susceptibility to *A. simplex*, our study suggests that this axis may represent a shared mechanistic pathway underlying vulnerability to intestinal opportunistic infections and a promising biomarker and therapeutic target in CD [4].

## Figures and Tables

**Table 1:** Demographic and clinical characteristics of patients with Crohn’s disease.

	*Encephalitozoon cuniculi*		
	Positive	Negative	Significance
Age	37.3 ± 14.5	41.7 ± 14.3	ns
HBI	7.7 ± 4.1	6.4 ± 3.5	ns
CDAI	191.3 ± 108.7	138.1 ± 107.9	ns
ESR	45.6 ± 41.4	32.1 ± 32.1	ns
CRP	48.9 ± 50.5	33.5 ± 14.6	ns
	n (%)	n (%)	
Gender			Odds Ratio
- Female	13 (50.0)	13 (50.0)	ns
- Male	10 (43.5)	14 (51.9)	
Clinical Scenarios			
- Active disease	11 (47.8)	12 (44.4)	ns
- New Patient	7 (30.4)	5 (18.5)	
- Remission	5 (21.7)	10 (37.0)	
Evolution			
- Inflammatory	12 (52.2)	17 (63.0)	ns
- Estenotic	7 (30.4)	7 (25.9)	
- Fistula	4 (17.4)	3 (11.1)	
Location			
- Ileum	12 (52.2)	14 (51.9)	ns
- Ileocolic	10 (43.5)	10 (37.0)	
- Colon	1 (4.3)	3 (11.1)	
Treatment			
- Yes	14 (77.8)	17 (70.8)	ns
- No	4 (22.2)	7 (29.2)	

HBI (Harvey-Bradshaw Index); CDAI (Crohn’s Disease Activity Index); ESR (Erythrocyte Sedimentation Rate); CRP (C-Reactive Protein); ns: not significant.

## Data Availability

The original contributions presented in this study are included in the article. Further inquiries can be directed to the corresponding authors.

## Supplementary Materials

The following supporting information can be downloaded at: https://www.mdpi.com/article/10.3390/pathogens15040429/s1, Figure S1: Gating strategy in healthy subjects vs. patients with Crohn disease; Figure S2: Western blot of caspase-3.

## Appendix A

**Table A1:** IL-7 gene and protein expression and Caspase-3 protein levels in Crohn’s disease and control samples. Protein expression values are expressed in arbitrary units (A.U.), corresponding to densitometric quantification of gel bands. Gene expression values are expressed as fold change relative to GAPDH, calculated using the 2^−ΔΔCt^ method.

Group	Sample	IL-7 Gene Expression	Caspase-3 Protein Expression	IL-7 Protein Expression
Crohn’s Disease	1	0.69	0.445	1.0
Crohn’s Disease	2	0.6	0.45	0.58
Crohn’s Disease	3	0.5	0.465	0.65
Crohn’s Disease	4	0.52	0.48	0.7
Crohn’s Disease	5	0.51	0.44	0.83
Crohn’s Disease	6	0.77	0.42	0.91
Crohn’s Disease	7	0.25	0.41	0.43
Crohn’s Disease	8	0.82	0.51	0.42
Crohn’s Disease	9	1.0	0.53	0.81
Control	10	0.0	0.59	0.15
Control	11	0.0	0.495	0.09
Control	12	0.0	0.55	0.13
Control	13	0.0	0.625	0.08
Control	14	0.0	0.655	0.08
Control	15	0.35	0.5	0.55
Control	16	0.0	0.61	0.14
Control	17	0.0	0.68	0.11
Control	18	0.0	0.74	0.12
Control	19	0.0	1.16	0.09
Control	20	0.48	1.09	0.45
Control	21	0.0	1.06	NA

NA: Not Available.

**Table A2:** CD127 (IL7Rα) and CD132 (IL7Rγ) gene expression in Crohn’s disease and control samples. Gene expression values are expressed as fold change relative to GAPDH, calculated using the 2^−ΔΔCt^ method.

Group	Sample	CD127 (IL7Rα) Gene Expression	CD132 (IL7Rγ) Gene Expression
Crohn’s Disease	1	2.8	0.4
Crohn’s Disease	2	3.56	0.88
Crohn’s Disease	3	3.88	1.04
Crohn’s Disease	4	3.84	0.72
Control	17	3.32	4.0
Control	18	4.0	4.4
Control	19	3.96	4.08
Control	20	3.68	4.06

**Table A3:** IL-7 and Caspase-3 Expression in Crohn’s Disease and Control Samples. Data are expressed as mean ± SD. Statistical comparisons were performed using unpaired Student’s *t*-test with Welch correction.

Variable	Crohn’s Disease (Mean ± SD, n)	Control (Mean ± SD, n)	*p*-Value
IL-7 Gene Expression (fold change vs. GAPDH)	0.629 ± 0.219 (n = 9)	0.069 ± 0.164 (n = 12)	<0.001
Caspase-3 Protein Expression (A.U.)	0.461 ± 0.040 (n = 9)	0.730 ± 0.237 (n = 12)	0.002
IL-7 Protein Expression (A.U.)	0.703 ± 0.203 (n = 9)	0.181 ± 0.161 (n = 11)	<0.001

**Table A4:** IL-7 Receptor Gene Expression (CD127 and CD132). Gene expression values are expressed as fold change vs. GAPDH (2^−ΔΔCt^). Data are mean ± SD. Statistical comparisons were performed using unpaired Student’s *t*-test with Welch correction.

Variable	Crohn’s Disease (Mean ± SD, n)	Control (Mean ± SD, n)	*p*-Value
CD127 (IL7Rα) Gene Expression	3.520 ± 0.501 (n = 4)	3.740 ± 0.314 (n = 4)	0.490
CD132 (IL7Rγ) Gene Expression	0.760 ± 0.273 (n = 4)	4.135 ± 0.180 (n = 4)	<0.001

**Table A5:** Anti-*Encephalitozoon cuniculi* optical density (O.D.) values according to treatment status. Data are expressed as mean ± standard deviation (SD). Statistical comparisons were performed using the unpaired Student’s *t*-test.

Variable	Treatment (Mean ± SD, n = 31)	NO (Mean ± SD, n = 19)	*p*-Value
IgG	1.4 ± 0.6	1.3 ± 0.5	0.738
IgM	0.6 ± 0.3	0.6 ± 0.4	0.418
IgA	0.8 ± 0.4	0.8 ± 0.4	1.0
IgE	0.04 ± 0.4	0.04 ± 0.4	0.958

## References

[1] Andreu-Ballester J.C., Pérez-Griera J., Garcia-Ballesteros C., Amigo V., Catalán-Serra I., Monforte-Albalat A., Bixquert-Jiménez M., Ballester F. (2013). Deficit of interleukin-7 in serum of patients with Crohn’s disease. Inflamm. Bowel Dis..

[2] Catalan-Serra I., Sandvik A.K., Bruland T., Andreu-Ballester J.C. (2017). Gammadelta T cells in Crohn’s disease: A new player in the disease pathogenesis?. J. Crohn’s Colitis.

[3] Andreu-Ballester J.C., Garcia-Ballesteros C., Amigo V., Ballester F., Gil-Borrás R., Catalán-Serra I., Magnet A., Fenoy S., Del Aguila C., Ferrando-Marco J. (2013). Microsporidia and its relation to Crohn’s disease: A retrospective study. PLoS ONE.

[4] Andreu-Ballester J.C., Hurtado-Marcos C., García-Ballesteros C., Pérez-Griera J., Izquierdo F., Ollero D., Jiménez A., Gil-Borrás R., Llombart-Cussac A., López-Chuliá F. (2025). Decreased gene expression of interleukin 2 receptor subunit γ (CD132) in tissues of patients with Crohn’s disease. World J. Gastroenterol..

[5] Hussain M.S., Bisht A.S., Gupta G. (2025). Reduced interleukin-2 receptor subunit γ expression in Crohn’s disease: A potential mechanism for γδ T cell deficiency. World J. Gastroenterol..

[6] Cuéllar C., Hurtado-Marcos C., Valdivieso E., Vaccaro L., González-Fernández J., Jiménez A.I., Pérez-Griera J., Benlloch S., Amorós C., Gil-Borrás R. (2026). Immune dysregulation, apoptosis impairment, and enhanced seroreactivity to Anisakis simplex in Crohn’s disease: Interplay of IL-7/IL-7R signalling and CD132 deficiency. Mem. Inst. Oswaldo Cruz.

[7] Schmidt E.C., Shadduck J.A. (1984). Mechanisms of resistance to the intracellular protozoan *Encephalitozoon cuniculi* in mice. J. Immunol..

[8] Khan I.A., Schwartzman J.D., Kasper L.H., Moretto M. (1999). CD8+ CTLs are essential for protective immunity against *Encephalitozoon cuniculi* infection. J. Immunol..

[9] Khan I.A., Moretto M., Weiss L.M. (2001). Immune response to *Encephalitozoon cuniculi* infection. Microbes Infect..

[10] Moretto M., Durell B., Schwartzman J.D., Khan I.A. (2001). Gamma delta T cell-deficient mice have a down-regulated CD8+ T cell immune response against *Encephalitozoon cuniculi* infection. J. Immunol..

[11] Moretto M., Weiss L.M., Khan I.A. (2004). Induction of a rapid and strong antigen-specific intraepithelial lymphocyte response during oral *Encephalitozoon cuniculi* infection. J. Immunol..

[12] Moretto M.M., Harrow D.I., Khan I.A. (2015). Effector CD8 T cell immunity in microsporidial infection: A lone defense mechanism. Semin. Immunopathol..

[13] Braunfuchsová P., Salát J., Kopecký J. (2001). CD8+ T lymphocytes protect SCID mice against *Encephalitozoon cuniculi* infection. Int. J. Parasitol..

[14] Han Y., Gao H., Xu J., Luo J., Han B., Bao J., Pan G., Li T., Zhou Z. (2020). Innate and adaptive immune responses against microsporidia infection in mammals. Front. Microbiol..

[15] Moretto M.M., Khan I.A., Weiss L.M., Becnel J.J. (2022). Immune response to microsporidia. Microsporidia.

[16] Goodman T., Lefrançois L. (1988). Expression of the gamma-delta T-cell receptor on intestinal CD8+ intraepithelial lymphocytes. Nature.

[17] Bonneville M., Janeway C.A., Ito K., Haser W., Ishida I., Nakanishi N., Tonegawa S. (1988). Intestinal intraepithelial lymphocytes are a distinct set of gamma delta T cells. Nature.

[18] Fischer M.A., Golovchenko N.B., Edelblum K.L. (2020). γδ T cell migration: Separating trafficking from surveillance behaviors at barrier surfaces. Immunol. Rev..

[19] Cuéllar C., Rodero M., Pérez-Griera J., Galindo-Regal L., Lopez-Chulia F., García-Ballesteros C., Andreu-Ballester J.C. (2022). Association between anti-Anisakis simplex antibodies and interleukin-7 levels. Int. Immunopharmacol..

[20] Lennard-Jones J.E. (1989). Classification of inflammatory bowel disease. Scand. J. Gastroenterol..

[21] Papay P., Ignjatovic A., Karmiris K., Amarante H., Milheller P., Feagan B., D’HAens G., Marteau P., Reinisch W., Sturm A. (2013). Optimising monitoring in the management of Crohn’s disease: A physician’s perspective. J. Crohn’s Colitis.

[22] del Aguila C., Rueda C., De la Camara C., Fenoy S. (2001). Seroprevalence of anti-Encephalitozoon antibodies in Spanish immunocompetent subjects. J. Eukaryot. Microbiol..

[23] Daschner A., Cuéllar C., Sánchez-Pastor S., Pascual C.Y., Martín-Esteban M. (2002). Gastro-allergic anisakiasis as a consequence of simultaneous primary and secondary immune response. Parasite Immunol..

[24] Gutiérrez R., Cuéllar C. (2002). Immunoglobulins anti-Anisakis simplex in patients with gastrointestinal diseases. J. Helminthol..

[25] Zeng Z., Mao H., Lei Q., He Y. (2025). IL-7 in autoimmune diseases: Mechanisms and therapeutic potential. Front. Immunol..

[26] Okada E., Yamazaki M., Tanabe M., Takeuchi T., Nanno M., Oshima S., Okamoto R., Tsuchiya K., Nakamura T., Kanai T. (2005). IL-7 exacerbates chronic colitis with expansion of memory IL-7Rhigh CD4+ mucosal T cells in mice. Am. J. Physiol. Gastrointest. Liver Physiol..

[27] Willis C.R., Seamons A., Maxwell J., Treuting P.M., Nelson L., Chen G., Phelps S., Smith C.L., Brabb T., Iritani B.M. (2012). Interleukin-7 receptor blockade suppresses adaptive and innate inflammatory responses in experimental colitis. J. Inflamm..

[28] Hasani Z., Aghdaei H.A., Balaii H., Azimirad M., Mirsamadi E.S., Mirjalali H., Zali M. (2017). The first study on opportunistic intestinal microsporidiosis in IBD patients receiving immunosuppressive medications in Iran. Epidemiol. Infect..

[29] Jin J., Tang Y., Cao L., Wang X., Chen Y., An G., Zhang H., Pan G., Bao J., Zhou Z. (2024). Microsporidia persistence in host impairs epithelial barriers and increases chances of inflammatory bowel disease. Microbiol. Spectr..

[30] Capitini C.M., Chisti A.A., Mackall C.L. (2009). Modulating T-cell homeostasis with IL-7: Preclinical and clinical studies. J. Intern. Med..

[31] Jacobs S.R., Michalek R.D., Rathmell J.C. (2010). IL-7 is essential for homeostatic control of T cell metabolism in vivo. J. Immunol..

[32] Belarif L., Danger R., Kermarrec L., Nerrière-Daguin V., Pengam S., Durand T., Mary C., Kerdreux E., Gauttier V., Kucik A. (2019). IL-7 receptor influences anti-TNF responsiveness and T cell gut homing in inflammatory bowel disease. J. Clin. Investig..

