# Supplementary figures and images for: Dysregulated IL-7/IL-7R-CD132 Axis and Intestinal Microsporidiosis in Crohn’s Disease
Source: Pathogens. 2026 Apr 16;15(4):429. doi: 10.3390/pathogens15040429 (PMC13118896; doi:10.3390/pathogens15040429)

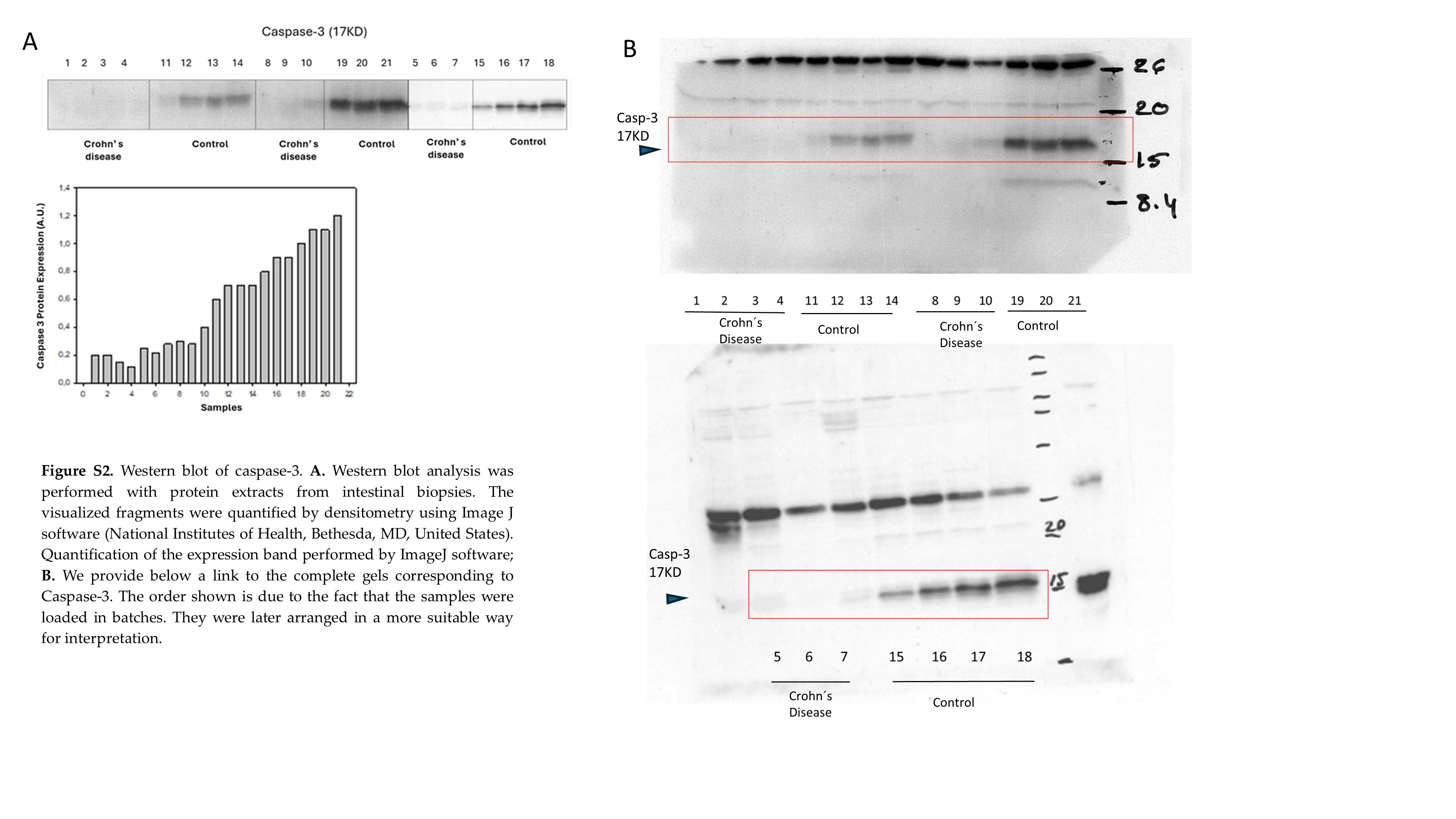

Supplement: Supplementary file 1 [file pathogens-15-00429-s001.zip › Figure S2.tif]

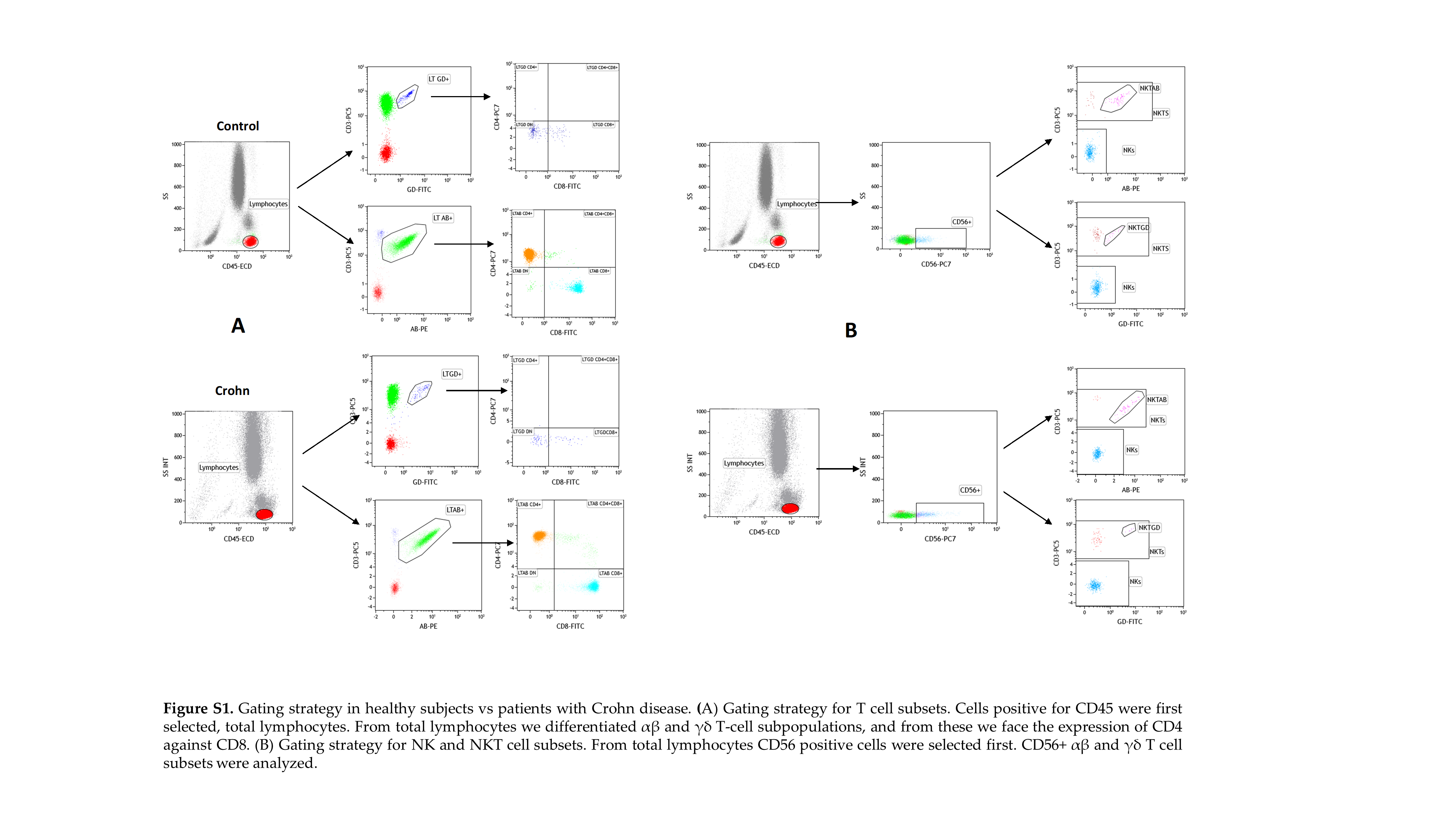

Supplement: Supplementary file 1 [file pathogens-15-00429-s001.zip › Figure S1.tif]
